# Quantitative Proteomic Analysis of Biogenesis-Based Classification for Extracellular Vesicles

**DOI:** 10.3390/proteomes8040033

**Published:** 2020-11-06

**Authors:** Linwen Zhang, Jeremie Parot, Vincent A. Hackley, Illarion V. Turko

**Affiliations:** 1 Biomolecular Measurement Division, National Institute of Standards and Technology, Gaithersburg, MD 20899, USA; lwzhang05@gmail.com; 2 Institute for Bioscience and Biotechnology Research, Rockville, MD 20850, USA; 3 Materials Measurement Science Division, National Institute of Standards and Technology, Gaithersburg, MD 20899, USA; jeremie.parot@gmail.com (J.P.); vince.hackley@nist.gov (V.A.H.); 4 Theiss Research, La Jolla, CA 92037, USA

**Keywords:** extracellular vesicles, exosomes, microvesicles, classification, targeted proteomics, QconCATs, multiple reaction monitoring, AF4

## Abstract

Extracellular vesicles (EVs) are traditionally divided into two major groups: (i) large vesicles originating from plasma membrane and called microvesicles, and (ii) small vesicles originating from the endoplasmic membrane and called exosomes. However, it is increasingly clear that the actual composition of a particular EV preparation cannot be adequately described with these two simple terms and is much more complex. Since the cell membrane origin of EVs predetermines their biological functions, the understanding of EV biogenesis is important for accurate interpretation of observed results. In the present study, we propose to take advantage of selective expression of some proteins in plasma or endosomal membranes and to use these proteins as plasma membrane-specific or endosomal membrane-specific markers. We have demonstrated that a quantitative mass spectrometry analysis allows simultaneous measurement of plasma membrane-specific and endosomal membrane-specific proteins in microvesicles and exosomes obtained after differential ultracentrifugation. Before mass spectrometry analysis, we also used sonicated platelets as a model of mixed EVs and multidetector asymmetrical-flow field-flow fractionation as an analytical method to verify a possible cross contamination of obtained microvesicles and exosomes. Based on the quantitative appearance of membrane-specific protein markers in EV preparations from human plasma and from human ARPE-19 cell medium, we concluded that there is no actual size limitation and both microvesicles and exosomes can be represented by large and small vesicles.

## 1. Introduction

Extracellular vesicles (EVs) comprise a heterogeneous class of nanosized membrane vesicles released from all cell types and play an essential role in intercellular communication [1]. EVs are a promising source of disease biomarkers [2,3,4] and have strong potential for use as site-specific drug carriers with fewer off-target side effects [5,6]. However, the classification of EVs has not been standardized [7]. Historically, more than a dozen terms based on biogenesis and size have been used to define EVs [8]. This long and confusing list was recently narrowed to a few major groups, including microvesicles and exosomes as the most common [9]. Microvesicles are typically defined as EVs with sizes from (100 to 1000) nm and formed by outward budding from the plasma membrane, while exosomes are often defined as EVs with sizes from (30 to 150) nm and formed by inward budding of the endosomal membrane [1,8,9,10,11]. Because of a generalization that microvesicles are large while exosomes are small, these EVs have commonly been separated by differential ultracentrifugation, where the particle fraction obtained by centrifugation (up to 20,000× g_n_) is called microvesicles. The particle fraction obtained by subsequent ultracentrifugation (106,000× g_n_ or higher) is then called exosomes. Although an obvious simplification, the microvesicles and exosome terms remain broadly used, while it is becoming clear that the overall picture is much more complex because of the fusion/rebound of multivesicular bodies with the plasma membrane. In addition, new mechanisms of EV biogenesis in various pathological conditions were proposed in recent years [1,8,9,10,11,12,13,14,15]. This addition made the field of EV biogenesis even more challenging with a particular interest in isolation and analysis of EV subpopulations [16,17]. Reasonably assuming that biogenesis predetermines biological properties of EVs, the analytical approaches to assign EV biogenesis are specifically attracting much attention.

Multiple attempts of a total proteomic analysis have been made to identify protein markers enriched in the specific EV subpopulations [18,19]. However, using a total proteomic analysis has a pitfall. The proteins identified as markers fall in a category of abundant cellular proteins and don’t have a unique cellular localization. In other words, these proteins can play the role of markers for particular EV subpopulations, but don’t identify the origin or biogenesis of these subpopulations. In the present work, we capitalized on the fact that the pattern of membrane proteins, in plasma and endosomal membranes, is rather different; there are proteins present only in plasma membrane (plasma membrane-specific) as well as proteins located mainly at the endosomal membrane (endosomal membrane-specific). Instead of total proteome analysis, we focused on a small group of rigorously selected proteins specifically associated with either plasma or endosomal membrane and used targeted proteome analysis based on multiple reaction monitoring (MRM) mass spectrometry [20,21] to quantify these proteins in preparations of microvesicles and exosomes.

Before MRM analysis, it was important to verify the possibility of cross contamination of microvesicles and exosomes and it was achieved by using sonicated platelets as an EV model and asymmetrical-flow field-flow fractionation (AF4) as a method of analysis. AF4 has been widely used to characterize nanoparticles and macromolecules [22,23], and has been recently tested for EV analysis [19,24,25,26]. Superior performance of AF4 in EV analysis is in part based on avoiding a solid phase interaction. In AF4, analytes are separated based on their hydrodynamic properties as they move through a thin ribbon-like channel under the influence of a cross-flow field applied through a semipermeable membrane.

Overall, three different biological sources, namely sonicated human platelets, human plasma, and ARPE-19 cell medium were used to obtain microvesicles and exosomes. MRM quantification in these samples was performed using four ^15^N-labeled quantification concatamers (QconCATs) as internal standards [27,28]. Taken together, the proteomic patterns of microvesicle and exosome preparations permitted us to evaluate their membrane origin.

## 2. Materials and Methods

### 2.1. ^15^N -Labeled QconCAT Expression, Purification, and Characterization

The amino acid sequences of GP1, GP2, PLout, and PLin QconCATs are shown in Figure S1 (Supplementary Materials). These QconCATs were designed for a broad EV analysis and carry the ability to quantify a total of 31 proteins. However, only 15 of them meet the criteria of being plasma membrane-specific or endosomal membrane-specific. The list of plasma membrane-specific proteins quantified in the present research includes integrin alpha-IIb, integrin beta-3, platelet glycoprotein Ib alpha (GP Ib alpha), platelet glycoprotein Ib beta (GP Ib beta), platelet glycoprotein V (GP V), platelet glycoprotein IX (GP IX), P2Y purinoceptor 1, P2Y purinoceptor 12, EH domain-containing protein 4 (EHD4), and low-affinity IgG Fc receptor II-a (IgG Fc receptor II-a). The list of endosomal membrane-specific proteins includes integrin alpha-2, P-selectin, cytochrome P-450 5A1 (CYP5A1), cyclooxygenase 2 (COX-2), and high-affinity IgG Fc receptor I (IgG Fc receptor I). The synthetic genes encoding these sequences were synthesized and incorporated into the pET21a expression vector with codon optimization for *E. coli* expression (Biomatik, Cambridge, ON, Canada). The expression, purification, and characterization of GP1, GP2, PLout, and PLin ^15^N-labeled QconCATs was performed as described before for other QconCATs [29]. Three optimal MRM transitions per each Q-peptide were experimentally selected after the tryptic digestion of QconCATs and summarized in Table S1 (Supplementary Materials).

### 2.2. Preparation of Microvesicles and Exosomes

Three biological samples were used to prepare and analyze microvesicles and exosomes. They include human platelets, human plasma, and human adult retinal pigment epithelial cell line-19 (ARPE-19). Pooled male platelets K2EDTA and pooled male plasma K2EDTA were obtained from BioreclamationIVT, Westbury, NY, USA.

ARPE-19 cells and two additional cell lines used in control experiments, human embryonic kidney (HEK-293) cells and human neuroblastoma (BE2) cells, were obtained from ATCC, Manassas, VA, and cultured following the manufacturer’s recommended protocols, https://www.atcc.org/en/Products/All/CRL-2302.aspx, https://www.atcc.org/products/all/crl-1573.aspx#, and https://www.atcc.org/Products/All/CRL-2268.aspx#, respectively. Medium to isolate EVs was collected when cells reached approximately 80% of confluence.

Human platelets collected from 50 mL of whole blood were resuspended in 2 mL of phosphate buffered saline (PBS) and sonicated with 10 pulses at duty cycle 20% and output control 2 using a Branson Sonifier 450 equipped with micro tip. For initial processing, sonicated platelets were centrifuged at 2000× g_n_ for 15 min to collect the supernatant. This step was repeated once, and the supernatant was subjected to 20,000× g_n_ centrifugation for 30 min. The pellet was dissolved in 1.0 mL of PBS and centrifuged again at 20,000× g_n_ for 30 min. The PBS-washed pellet was re-dissolved in 0.2 mL of PBS and called the 20K pellet. The 20K pellet is a representative sample of microvesicles. The supernatant of 20,000× g_n_ centrifugation was further centrifuged at 106,000× g_n_ for 60 min using a Beckman TLA-55 rotor and TL-100 ultracentrifuge. The pellet was dissolved in 1.0 mL of PBS and centrifuged again at 106,000× g_n_ for 60 min. The PBS-washed pellet was redissolved in 0.2 mL of PBS and called the 106K pellet. The 106K pellet is a representative sample of exosomes.

Typical isolation of EVs from human plasma or ARPE-19 cell medium started with 200 mL of plasma or 500 mL of medium. Isolations for HEK-293 and BE2 cells medium were performed from 50 mL of medium. The centrifugation protocols were the same as described for sonicated platelets and also generated 20K and 106K pellet samples.

### 2.3. AF4

20K and 106K pellet samples from sonicated platelets were further fractionated using AF4. We used an Eclipse DualTec (Wyatt Technology, Santa Barbara, CA, USA) equipped with a degasser (Gastorr TG-14, Flom Co., Ltd., Tokyo, Japan), 1100-series isocratic pump (Agilent Technologies, Santa Clara, CA, USA), 1260 ALS series autosampler (Agilent Technologies, Santa Clara, CA, USA), 1200 series UV-vis absorbance diode array detector (Agilent Technologies, Santa Clara, CA, USA), and a DAWN HELEOS II multiangle light scattering detector with QELS operating at a laser wavelength of 661 nm (Wyatt Technology, Santa Barbara, CA, USA). The AF4 conditions and method are provided in Table S2 (Supplementary Materials).

### 2.4. LC-MS/MS Analysis

The protein samples in PBS were supplemented with 2% sodium dodecyl sulfate and 2 µL was used for total protein concentration determination with a Nanodrop 2000. Samples were then supplemented with GP1, GP2, PLout, and PLin ^15^N-labeled QconCATs (5 pmol each). The following alkylation with iodoacetamide and digestion with trypsin in the presence of 0.1% RapiGest was performed as described before [29]. MRM analysis of tryptic peptides was performed on an Agilent 6490 iFunnel Triple Quadrupole LC/MS system (Santa Clara, CA, USA) as described before in detail [29].

### 2.5. Data Analysis

Every transition measured per peptide was taken as an individual measurement. MRM peak area integration was performed using Skyline (University of Washington, Washington, DC, USA). The peak ratios from thee transitions per peptide were averaged to yield the peptide ratios. All experiments were performed in duplicate and data are represented as the mean ± SD (*n* = 6).

## 3. Results and Discussion

### 3.1. Assignment of Protein Markers

The first step to investigate a biogenesis of EVs in the 20K and 106K pellets was to assign protein markers for plasma and endosomal membranes. Unfortunately, well-known EVs membrane proteins that we used to quantify in previous studies [29,30], such as TSG101, moesin, ADAM10, L1CAM and flotillin-1, can be found in both plasma and intracellular membranes and cannot be used in the present study. Another group of established EVs membrane proteins called tetraspanins (CD9, CD63, and CD81) are short proteins with four transmembrane segments and don’t have tryptic peptides suitable for MRM assay. We decided to use the Human Protein Atlas (https://www.proteinatlas.org/) and the UniProt website (https://www.uniprot.org/) to find the selective protein markers for plasma and endosomal membranes. Additional criteria of our search was availability of several tryptic peptides per protein suitable for MRM assay. Since we planned to use human plasma as a source of EVs, we also focused on platelet proteins because it is generally believed that platelets are the major source of EVs in circulation [31,32,33].

The final list of selected protein markers included 10 plasma membrane-specific proteins (integrin alpha-IIb, integrin beta-3, platelet glycoprotein Ib alpha, platelet glycoprotein Ib beta, platelet glycoprotein V, platelet glycoprotein IX, P2Y purinocepter 1, P2Y purinocepter 12, EHD4, and IgG Fc receptor II-a) and five endosomal membrane-specific proteins (integrin alpha-2, P-selectin, CYP5A1, COX-2, and IgG Fc receptor I).

### 3.2. Sonicated Platelets

Sonication of platelets in vitro is an artificial way to generate membrane vesicles. We used it as a model to address two question: (i) whether there is a cross-contamination of 20K and 106K pellets obtained by differential ultracentrifugation, and (ii) whether there is any physical limitation (something associated with curvature or mechanics of membrane) that predetermines generating smaller size vesicles from endosomal membrane in comparison to plasma membrane. We used a soft sonication to avoid extensive fragmentation of both membranes.

**Figure 1:**
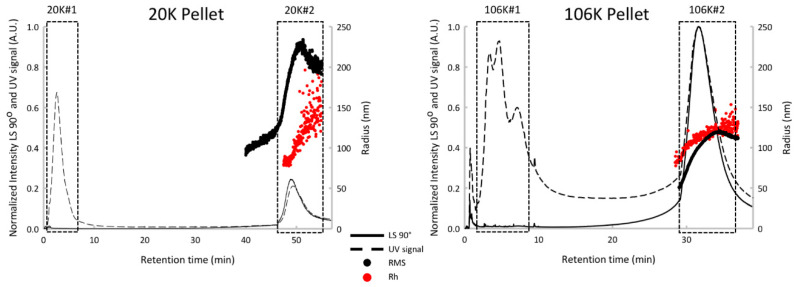
AF4 fractograms of 20K and 106K pellets from sonicated platelets. Protein peak #1 and vesicle peak #2 are marked with rectangles. The strong UV absorbance at short retention times and overlapping the void volume is attributed to eluting proteins separated from the original centrifuged precipitates. AF4 elution is reversed relative to size exclusion chromatography with smaller particles eluting faster. RMS is the root-mean-square radius. R_h_ is the hydrodynamic radius.

To assess a possible cross-contamination of vesicles in both pellets, the AF4 has been used. AF4 only recently attracted attention following the report of a successful separation of EVs into subpopulations [19,34]. We have to mention here that because of sample dilution during the analysis, current applications of AF4 have a limitation when the EV sample is too small. The advantage of using sonicated platelets as a model EV sample was in an unlimited amount of available sonicated platelets. As it was expected [19,34], the bulk protein was found in peak #1 and separated from vesicle-associated proteins eluting in peak #2 for both 20K and 106K pellets (Figure 1). The important observation was that the vesicles peaks from 20K and 106K pellets are well resolved with retention time at a peak maximum of 49 min and 31 min, respectively (Figure 1) and at least in term of AF4 sensitivity do not cross contaminate. In other words, the 20K pellet was represented by a single vesicles peak at 49 min while the 106K pellet was represented by a single vesicles peak at 31 min.

In addition, the multidetector-AF4 approach offers a detailed characterization. The 20K vesicles peak is characterized by a mean hydrodynamic radius (*R*_h_) at 191 nm (±26 nm), an average root-mean-square radius (RMS) at 191 nm (±30 nm), a shape factor (RMS/*R*_h_) of 1.0 and a mass fraction of around 36%. The 106K vesicles peak is characterized by *R*_h_ at 118 nm (±6 nm), a RMS at 96 nm (±16 nm), a shape factor of 0.81 and a mass fraction of around 40%. The 20K pellet is composed of larger vesicles compared with the 106K pellet, with a shape factor consistent with thin hollow spheres (1.0), while 106K is characterized by a shape factor associated with solid spheres (0.78). Using these multidetector-AF4 results, we can highlight the presence of two vesicles populations presenting specific physicochemical properties between the two pellets. This confirms the efficacy of using differential ultracentrifugation in preparing 20K and 106K pellets with very little (if any) cross contamination.

**Figure 2:**
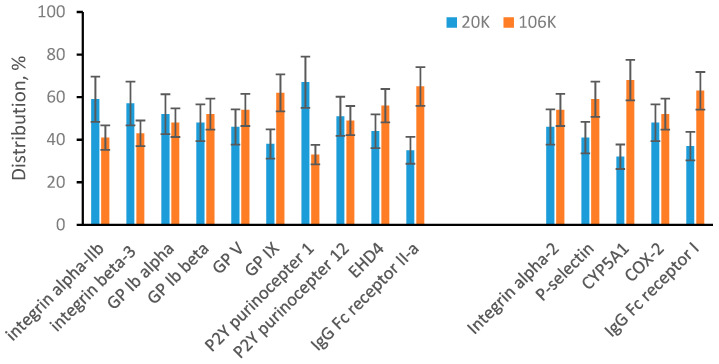
MRM analysis of 20K and 106K pellets from sonicated platelets. Plasma membrane-specific and endosomal membrane-specific proteins are arranged in two separated groups. Bar graphs show a percentile distribution of each listed protein between 20K and 106K pellets. Data are represented as the mean ± SD (*n* = 6).

The MRM quantification data are summarized in Figure 2. The data were plotted into two groups of protein markers: plasma membrane-specific and endosomal membrane-specific. The bar graphs show a percentile distribution of a specific protein between 20K pellet (blue bars) and 106K pellet (red bars). Plasma membrane-specific proteins show approximately 50/50 distribution between the 20K and 106K pellets. It means that after soft sonication, plasma membrane-specific proteins are about evenly distributed between large vesicles (20K pellet) and small vesicles (106K pellet). For endosomal membrane-specific proteins, the range of distribution is broader, and some proteins show preference to either large or small vesicles. For example, approximately 68% of CYP5A1 was found in small vesicles (106K pellet). However, overall endosomal membrane-specific proteins are present at high concentrations in both large (20K pellet) and small (106K pellet) vesicles. In summary, we concluded that there is no physical limitation and both membranes, plasma and endosomal, can generate both large and small vesicles.

### 3.3. EVs from Human Plasma

**Figure 3:**
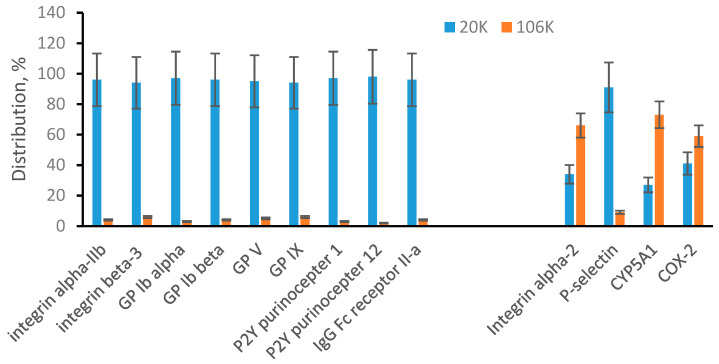
Multiple reaction monitoring (MRM) analysis of microvesicles and exosomes from plasma. Plasma membrane-specific and endosomal membrane-specific proteins are arranged in two separated groups. Bar graphs show a percentile distribution of each listed protein between microvesicles (20K) and exosomes (106K). Data are represented as the mean ± SD (*n* = 6).

Plasma is a body fluid commonly used as the source of EVs. We were able to quantify 9 plasma membrane-specific proteins and all of them were preferably found in 20K pellet (Figure 3). This suggests that plasma microvesicles are mainly large particles that appear in 20K pellet. At the same time, 4 endosomal membrane-specific proteins were found distributed between 20K and 106K pellet samples, including P-selectin found preferably in 20K pellet. Integrin alpha-2, and CYP5A1 were found preferably in 106K pellet while COX-2 was approximately evenly distributed between 20K and 106K pellets. We concluded that plasma exosomes can be both large and small and appear in both 20K and 106K pellets. Our targeted proteomic quantification is consistent with a published observation that suggests exosomes are a heterogeneous population of particles with a broad size range [10,17,34,35]. The interesting new observation is that P-selectin was found mainly in large exosomes (20K pellet). Preferable distribution of individual proteins in different EV subpopulations is an early observation for tetraspanins (such as CD9, CD63, and CD81) and under specific pathological conditions for some other proteins [10,17]. We show here that P-selectin could be an additional marker of an EV subpopulation. This also emphasizes the importance of further development of affinity-based methods for detecting, isolating, and targeting specific subpopulations of EVs.

### 3.4. EVs from ARPE-19 Cell Medium

**Figure 4:**
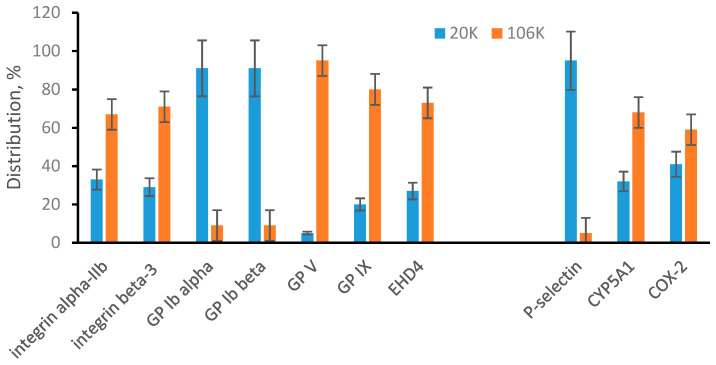
MRM analysis of microvesicles and exosomes from ARPE-19 cells. Plasma membrane-specific and endosomal membrane-specific proteins are arranged in two separated groups. Bar graphs show a percentile distribution of each listed protein between microvesicles (20K) and exosomes (106K). Data are represented as the mean ± SD (*n* = 6).

In addition to body fluids, the cell culture medium is another commonly used source of EVs. We have previous experience with cultivating ARPE-19 cells [36] and this was the reason for their selection as a representative cell culture medium source. An initial and unexpected observation was that the 20K pellet is very small in comparison to results from plasma. Based on total protein measurement for plasma samples, the 20K pellet was approximately 60% of the 106K pellet. For the ARPE-19 cell culture medium, the 20K pellet was not greater than 5% of 106K pellet. To determine if this is unique for ARPE-19 cells, we have performed the same type of differential ultracentrifugation and total protein measurement for two other cell culture media. The 20K pellet was not greater than 8% of 106K pellet for samples collected from human embryonic kidney (HEK-293) cells and from human neuroblastoma (BE2) cells. In total for all three cell lines, the 20K pellet was much smaller relative to the 106K pellet in comparison to the proportion observed in plasma. All cell lines were cultured in the presence of 10% fetal bovine serum. If we assume contamination of cell EVs with bovine serum EVs, we must also assume that the proportion of 20K EVs to 106K EVs in bovine serum is at least one order of magnitude different from the proportion in human plasma. MRM measurements show that this is not the case and cell EVs in our preparation were not contaminated with bovine EVs, at least on the level of MRM detection. MRM measurements were performed based on two peptides per protein. For the seven proteins shown in Figure 4, both peptides are unique for human proteins. However, for GP Ib beta, EHD4, and CYP5A1, one peptide is unique for human proteins while a second peptide is common for both human and bovine proteins (Table S1, Supplementary Materials). Since absolute quantifications of GP Ib beta, EHD4, and CYP5A1 were essentially identical based on individual peptides, we have concluded that cell EVs in our preparations are not contaminated with bovine EVs.

Quantification of plasma membrane-specific proteins in the 20K and 106K pellets from ARPE-19 medium revealed another difference in comparison to plasma (Figure 4). All seven detected plasma membrane-specific proteins were in some way distributed between 20K and 106K pellets. This points to the presence of large and small microvesicles in ARPE-19 medium and is different from plasma microvesicles that were mainly represented by large vesicles. Distribution of three detected endosomal membrane-specific proteins (Figure 4) is similar to those observed for plasma (Figure 3) with P-selectin found mainly in the 20K pellet and overall points to the presence of small and large exosomes.

## 4. Conclusions

We have demonstrated that an MRM assay for proteins specifically associated with plasma or endosomal membrane can help to track the biogenesis of EVs in a particular preparation. The concurrent application of AF4 offers a real-time in situ physicochemical characterization of each EV population and can be used for assessing possible cross contamination of EV subpopulations. With further development of AF4 methodologies and the ability to obtain fractions of EV subpopulations, MRM targeting of specific proteins can also help in identifying those that play a role as subpopulation-specific markers. In terms of biogenesis-based classification of EVs, this work concurs well with the International Society for Extracellular Vesicles (ISEV) recommendation [37] to avoid terms other than EVs unless a specific subpopulation of EVs is clearly isolated and characterized.

## Supplementary Materials

The following are available online at https://www.mdpi.com/2227-7382/8/4/33/s1, Table S1: Transitions used for quantification, Table S2: AF4 conditions and method, Figure S1: The amino acid sequences of GP1, GP2, PLin, and PLout QconCATs.

## Abbreviations

AF4	asymmetrical-flow field-flow fractionation
EV	extracellular vesicle
GP	glycoprotein
MRM	multiple reaction monitoring
QconCATs	quantification concatamer

## References

[1] Abels E.R., Breakefield X.O. (2016). Introduction to extracellular vesicles: Biogenesis, RNA cargo selection, content, release, and uptake. Cell. Mol. Neurobiol..

[2] Ludwig N., Whiteside T.L., Reichert T. (2019). Challenges in exosome isolation and analysis in health and disease. Int. J. Mol. Sci..

[3] Jiang L., Gu Y., Du Y., Liu J. (2019). Exosomes: Diagnostic biomarkers and therapeutic delivery vehicles for can-cer. Mol. Pharm..

[4] Roy S., Hochberg F.H., Jones P.S. (2018). Extracellular vesicles: The growth as diagnostics and therapeutics; a survey. J. Extracell. Vesicles.

[5] Gudbergsson J.M., Jonsson K., Simonsen J.B., Johnsen K.B. (2019). Systematic review of targeted extracellular vesicles for drug delivery—Consideration of methodological and biological heregeneity. J. Cont. Rel..

[6] Reiner A.T., Witwer K.W., Van Balkom B.W., De Beer J., Brodie C., Corteling R.L., Gabrielsson S., Gimona M., Ibrahim A.G., De Kleijn D. (2017). Concise review: Developing best-practice models for the therapeutic use of extracellular vesicles. STEM CELLS Transl. Med..

[7] Witwer K.W., Buzas E.I., Bemis L.T., Bora A., Lasser C., Lötvall J., Nolte-‘t Hoen E.N., Piper M.G., Sivaraman S., Skog J. (2013). Standardization of sample collection, iso-lation and analysis methods in extracellular vesicle research. J. Extracell. Vesicles.

[8] Akers J.C., Gonda D., Kim R., Carter B.S., Chen C.C. (2013). Biogenesis of extracellular vesicles (EV): Exosomes, microvesicles, retrovirus-like vesicles, and apoptotic bodies. J. Neuro-Oncol..

[9] Raposo G., Stoorvogel W. (2013). Extracellular vesicles: Exosomes, microvesicles, and friends. J. Cell Biol..

[10] Willms E., Cabañas C., Mäger I., Wood M.J.A., Vader P. (2018). Extracellular vesicle heterogeneity: Subpopulations, isolation techniques, and diverse functions in cancer progression. Front. Immunol..

[11] Latifkar A., Hur Y.H., Sanchez J.C., Cerione R.A., Antonyak M.A. (2019). New insights into extracellular vesicle biogenesis and function. J. Cell Sci..

[12] Busatto S., Zendrini A., Radeghieri A., Paolini L., Romano M., Presta M., Bergese P. (2019). The nanostruc-tured secretome. Biomater. Sci..

[13] Gudbergsson J.M., Johnsen K.B. (2019). Exosomes and autophagy: Rekindling the vesicular waste hypothesis. J. Cell Commun. Signal..

[14] McAndrews K.M., Kalluri R. (2019). Mechanisms associated with biogenesis of exosomes in cancer. Mol. Cancer.

[15] Battistelli M., Falcieri E. (2020). Appoptotic bodies: Particular extracellular vesicles involved in intercellular communication. Biology.

[16] Jang S.C., Crescitelli R., Cvjetkovic A., Belgrano V., Bagge R.O., Sundfeldt K., Ochiya T., Kalluri R., Lötvall J. (2019). Mitochondrial protein enriched extracellular vesicles discovered in human melanoma tissues can be detected in patient plasma. J. Extracell. Vesicles.

[17] Crescitelli R., Lässer C., Jang S.C., Cvjetkovic A., Malmhäll C., Karimi N., Höög J.L., Johansson I., Fuchs J., Thorsell A. (2020). Subpopulations of extracellular vesicles from human metastatic melanoma tissue identified by quantitative proteomics after optimized isolation. J. Extracell. Vesicles.

[18] Kowal J., Arras G., Colombo M., Jouve M., Morath J.P., Primdal-Bengtson B., Dingli F., Loew D., Tkach M., Thery C. (2016). Proteomic comparison defines novel markers to characterize heterogeneous popula-tions of extracellular vesicle subtypes. Proc. Nat. Acad. Sci. USA.

[19] Zhang H., Freitas D., Kim H.S., Fabijanick K., Li Z., Chen H., Mark M.T., Molina H., Martin A.B., Bojmar L. (2018). Identification of distinct nanoparticles and subsets of extracellular vesicles by assymetric flow field-flow fractionation. Nat. Cell Biol..

[20] Anderson L., Hunter C.L. (2006). Quantitative mass spectrometry multiple reaction monitoring assays for major plasma proteins. Mol. Cell. Proteom..

[21] Liebler D.C., Zimmerman L.J. (2013). Targeted quantification of proteins by mass spectrometry. Biochemistry.

[22] Williams S.K.R., Runyon J.R., Murtaza G. (2011). Field-flow fractionation: Addressing the nano challenge. Anal. Chem..

[23] Qureshi R.N., Kok W.T. (2011). Application of flow field-flow fractionation for the characterization of macro-molecules of biological interest: A review. Anal. Bioanal. Chem..

[24] Sitar S., Kejzar A., Pahovnik D., Kogej K., Tusek-Znidaric M., Lenassi M., Zagar E. (2015). Size characteriza-tion and quantification of exosomes by asymmetrical-flow field-flow fractionation. Anal. Chem..

[25] Yang J.S., Lee J.C., Byeon S.K., Rha K.H., Moon M.H. (2017). Size dependent lipidomic analysis of urinary exosomes from patients with prostate cancer by flow field-flow fractionation and nanoflow liquid chro-matography-tandem mass spectrometry. Anal. Chem..

[26] Oeyen E., Mol K.V., Baggerman G., Willems H., Boonen K., Rolfo C., Pauwels P., Jacobs A., Schil-dermans K., Cho W.C. (2018). Ultrafiltration and size exclusion chromatography combined with asymmetrical-flow field-flow fractionation for the isolation and characterization of extracellular vesicles from urine. J. Extracell. Vesicles.

[27] Pratt J.M., Simpson D.M., Doherty M.K., Rivers J., Gaskell S.J., Beynon R.J. (2006). Multiplexed absolute quan-tification for proteomics using concatenated signature peptides. Nat. Protoc..

[28] Chen J., Turko I.V. (2014). Trends in QconCATs for targeted proteomics. TrAC Trends Anal. Chem..

[29] Wang T., Anderson K.W., Turko I.V. (2017). Assessment of extracellular vesicles purity using proteomic stand-ards. Anal. Chem..

[30] Wang T., Turko I.V. (2018). Proteomic toolbox to standardize the separation of extracellular vesicles and lipo-protein particles. J. Proteome Res..

[31] Au A.E., Josefsson E.C. (2016). Regulation of platelet membrane protein shedding in health and disease. Platelets.

[32] Brisson A.R., Tan S., Linares R., Gounou C., Arraud N. (2017). Extracellular vesicles from activated platelets: A semiquantitative cryo-electron microscopy and immuno-gold labeling study. Platelets.

[33] Nielsen T., Kristensen A.F., Pedersen S., Christiansen G., Kristensen S.R. (2018). Investigation of procoagulant activity in extracellular vesicles isolated by differential ultracentrifugation. J. Extracell. Vesicles.

[34] Zhang H., Lyden D. (2019). Assymmetric-flow field-flow fractionation technology for exomere and small extra-cellular vesicle separation and characterization. Nat. Protoc..

[35] Simonsen J.B. (2017). What are we looking at? Extracellular vesicles, lipoproteins, or both?. Circ. Res..

[36] Liao W.-L., Turko I.V. (2009). Accumulation of large protein fragments in prematurely senescent ARPE-19 Cells. Investig. Opthalmol. Vis. Sci..

[37] Witwer K.W., Thery C. (2019). Extracellular vesicles or exosomes? On primacy, precision, and popularity influ-encing a choice of nomenclature. J. Extracell. Vesicles.

