# Supplementary material for: Quantitative Proteomic Analysis of Biogenesis-Based Classification for Extracellular Vesicles
Source: Proteomes. 2020 Nov 6;8(4):33. doi: 10.3390/proteomes8040033 (PMC7709127; doi:10.3390/proteomes8040033)
Supplement: Supplementary file 1 [file proteomes-08-00033-s001.pdf]

## Supplementary Material

# Quantitative proteomic analysis of biogenesis-based classification for extracellular vesicles

Linwen Zhang[a,b], Jeremie Parot[c,d], Vincent A. Hackley[c] and Illarion V. Turko[a,b,*]

[a]Biomolecular Measurement Division, National Institute of Standards and Technology, Gaithersburg, Maryland 20899, United States

[b]Institute for Bioscience and Biotechnology Research, Rockville, Maryland 20850, United States

[c]Materials Measurement Science Division, National Institute of Standards and Technology, Gaithersburg, Maryland 20899, United States

[d]Theiss Research, La Jolla, California 92037, United States

**Table S1.** Transitions used for quantification
**Table S2.** AF4 conditions and method
**Figure S1.** The amino acid sequences of GP1, GP2, PLin, and PLout QconCATs

**Table S1.** Transitions used for quantification

| Protein | Peptide | L/H | Precursor (m/z) | Product Ions (m/z) 1 | 2 | 3 |
|---|---|---|---|---|---|---|
| Integrin alpha-IIb | FGSAIAPLGDLDR | L | 666.4 | 688.4 (y6) | 785.4 (y7) | 856.5 (y8) |
| | | H | 674.3 | 697.3 (y6) | 795.4 (y7) | 867.4 (y8) |
| | GEAQVWTQLLR | L | 650.9 | 816.5 (y6) | 915.5 (y7) | 1043.6 (y8) |
| | | H | 659.3 | 827.4 (y6) | 927.5 (y7) | 1057.6 (y8) |
| Integrin beta-3 | VLEDRPLSDK | L | 586.3 | 559.3 (y5) | 715.4 (y6) | 830.4 (y7) |
| | | H | 593.3 | 565.3 (y5) | 725.4 (y6) | 841.4 (y7) |
| | GSGDSSQVTQVSPQR | L | 766.9 | 815.4 (y7) | 914.5 (y8) | 1042.6 (y9) |
| | | H | 777.3 | 827.4 (y7) | 927.5 (y8) | 1057.5 (y9) |
| GP Ib alpha | GLGELQELYLK | L | 631.9 | 665.4 (y5) | 793.4 (y6) | 906.5 (y7) |
| | | H | 638.3 | 671.4 (y5) | 801.4 (y6) | 915.5 (y7) |
| | GVLQGHLESSR | L | 591.8 | 728.4 (y6) | 785.4 (y7) | 913.4 (y8) |
| | | H | 600.3 | 739.3 (y6) | 797.4 (y7) | 927.4 (y8) |
| GP Ib beta | LSLTDPLVAER | L | 607.3 | 684.4 (y6) | 799.4 (y7) | 900.5 (y8) |
| | | H | 614.3 | 693.4 (y6) | 809.4 (y7) | 911.4 (y8) |
| | LLPYLAEDELR | L | 666.4 | 732.4 (y6) | 845.4 (y7) | 1008.5 (y8) |
| | | H | 673.3 | 741.3 (y6) | 855.4 (y7) | 1019.5 (y8) |
| GP V | SIAPGAFDR | L | 467.2 | 508.3 (y4) | 565.3 (y5) | 662.3 (y6) |
| | | H | 473.2 | 515.2 (y4) | 573.2 (y5) | 671.3 (y6) |
| | LPNLSSLTLSR | L | 600.9 | 676.4 (y6) | 763.4 (y7) | 876.5 (y8) |
| | | H | 608.3 | 685.4 (y6) | 773.4 (y7) | 887.5 (y8) |
| GP IX | GHGLTALPALPAR | L | 637.4 | 737.5 (y7) | 808.5 (y8) | 909.6 (y9) |
| | | H | 646.3 | 747.4 (y7) | 819.5 (y8) | 921.5 (y9) |
| | TPEALLQVR | L | 513.8 | 628.4 (y5) | 699.5 (y6) | 828.5 (y7) |
| | | H | 520.3 | 637.4 (y5) | 709.4 (y6) | 839.5 (y7) |
| P2Y purinoceptor 1 | YSGVVYPLK | L | 513.3 | 520.3 (y4) | 619.4 (y5) | 718.4 (y6) |
| | | H | 518.3 | 525.3 (y4) | 625.4 (y5) | 725.4 (y6) |
| | VYATYQVTR | L | 550.8 | 666.4 (y5) | 767.4 (y6) | 838.4 (y7) |
| | | H | 557.3 | 675.3 (y5) | 777.4 (y6) | 849.4 (y7) |
| P2Y purinoceptor 12 | IPYTLSQTR | L | 539.8 | 604.3 (y5) | 705.4 (y6) | 868.5 (y7) |
| | | H | 546.3 | 613.3 (y5) | 715.4 (y6) | 879.4 (y7) |
| | SNFIIFLK | L | 491.3 | 520.3 (y4) | 633.4 (y5) | 780.5 (y6) |
| | | H | 496.3 | 525.3 (y4) | 639.4 (y5) | 787.5 (y6) |
| EHD4 | SISVIDSPGILSGEK | L | 751.4 | 800.5 (y8) | 887.5 (y9) | 1002.5 (y10) |
| | | H | 759.4 | 809.4 (y8) | 897.5 (y9) | 1013.5 (y10) |
| | LDISDEFSEAIK | L | 683.8 | 547.3 (y5) | 694.4 (y6) | 823.4 (y7) |
| | | H | 690.3 | 553.3 (y5) | 701.4 (y6) | 831.4 (y7) |
| IgG Fc receptor II-a | AAQFEPPGR | L | 486.7 | 426.2 (y4) | 555.3 (y5) | 702.4 (y6) |
| | | H | 493.2 | 433.2 (y4) | 563.3 (y5) | 711.3 (y6) |
| | VTFFQNGK | L | 470.7 | 446.2 (y4) | 593.3 (y5) | 740.4 (y6) |
| | | H | 476.2 | 453.2 (y4) | 601.3 (y5) | 749.3 (y6) |
| Integrin alpha-2 | FVQGLDIGPTK | L | 587.8 | 630.3 (y6) | 743.4 (y7) | 800.5 (y8) |
| | | H | 594.3 | 637.3 (y6) | 751.4 (y7) | 809.4 (y8) |

| | | | | | | |
|---|---|---|---|---|---|---|
| | IGQTSSSVSFK | L | 570.8 | 654.3 (y6) | 741.4 (y7) | 842.4 (y8) |
| | | H | 577.3 | 661.3 (y6) | 749.4 (y7) | 851.4 (y8) |
| P-selectin | YTDLVAIQNK | L | 582.8 | 573.3 (y5) | 672.4 (y6) | 785.5 (y7) |
| | | H | 589.3 | 581.3 (y5) | 681.4 (y6) | 795.5 (y7) |
| | NEIDYLNK | L | 504.8 | 537.3 (y4) | 652.3 (y5) | 765.4 (y6) |
| | | H | 510.2 | 543.3 (y4) | 659.3 (y5) | 773.4 (y6) |
| CYP5A1 | DELNGFFNK | L | 542.3 | 612.3 (y5) | 726.4 (y6) | 839.4 (y7) |
| | | H | 548.2 | 619.3 (y5) | 735.3 (y6) | 849.4 (y7) |
| | QVLVENFSNFTNR | L | 784.4 | 885.4 (y7) | 999.5 (y8) | 1128.5 (y9) |
| | | H | 794.4 | 897.4 (y7) | 1013.4 (y8) | 1143.5 (y9) |
| COX-2 | TVTINASSSR | L | 518.3 | 507.3 (y5) | 621.3 (y6) | 734.4 (y7) |
| | | H | 525.3 | 515.2 (y5) | 631.3 (y6) | 745.3 (y7) |
| | VSQASIDQSR | L | 545.8 | 618.3 (y5) | 705.4 (y6) | 776.4 (y7) |
| | | H | 553.3 | 627.3 (y5) | 715.3 (y6) | 787.4 (y7) |
| IgG Fc receptor I | ITSASVNDSGEYR | L | 699.8 | 726.3 (y6) | 840.3 (y7) | 939.4 (y8) |
| | | H | 708.3 | 735.3 (y6) | 851.3 (y7) | 951.4 (y8) |
| | LVYNVLYYR | L | 601.8 | 713.4 (y5) | 827.4 (y6) | 990.5 (y7) |
| | | H | 608.3 | 721.4 (y5) | 837.4 (y6) | 1001.5 (y7) |

Transitions are listed for both unlabeled, light (L) and fully $^{15}$N-labeled, heavy (H) peptides. All precursors were +2 charge and product ions were +1 charge. Additionally, y-ion information is included.

**Table S2.** AF4 conditions and method

An Eclipse DualTec (Wyatt Technology, Santa Barbara, CA, USA) equipped with a degasser (Gastorr TG-14, Flom Co., Ltd, Tokyo, Japan), 1100-series isocratic pump (Agilent Technologies, Santa Clara, CA), 1260 ALS series autosampler (Agilent Technologies), 1200 series UV-vis absorbance diode array detector (Agilent Technologies), and a DAWN HELEOS II multi-angle light scattering detector with QELS operating at a laser wavelength of 661 nm (Wyatt Technology).

UV absorbance is measured at 280 nm using an extinction coefficient of 0.667 mL/(mg cm). The DAWN HELEOS is calibrated using toluene at the 90° detector, and the other 17 detectors are normalized using a Rayleigh scatterer (bovine serum albumin) in water. The 12-detector position is used for the online QELS detector providing DLS measurements at an angle of 99.9 º.

Fractionation experiments are conducted using the following experimental method: MALS cell temperature set at 25 °C, mobile phase consisting of phosphate buffered saline (PBS), short channel (145 mm length, Wyatt Technology) with a 350 µm trapezoidal mylar spacer, a 10 kDa (kg/mol) molecular weight cut-off regenerated cellulose membrane and an injection volume of 80 µL.

The fractionation method is summarized below in tabular format.

| Type of flow | Duration (min) | Cross-Flow or Focus flow (mL/min) | Elution flow (mL/min) |
|---|---|---|---|
| Elution | 3 | - | 0.5 |
| Focus | 2 | 1.5 | - |
| Focus + injection (0.2 mL/min) | 5 | 1.5 | - |
| Elution | 40 | 0.5-0.05<br>Linear decrease | 0.5 |
| Elution | 10 | 0 | 0.5 |

AF4 data is collected and analyzed using OpenLab (Agilent Technologies) and Astra 6.1.4.25 (Wyatt Technology) software.

**Figure S1.** The amino acid sequences of GP1, GP2, PLin, and PLout QconCATs

**GP1.** Number of amino acids: 585; Molecular weight: 64 670.02; Theoretical pI: 9.93; Grand average of hydropathicity (GRAVY): -0.327.

MSAIGLKIGDTVSFSIEAKVRGAPQEKEKSFTIKPVGFKDSLIVQTQLYGRFGSAIAPLG
DLDRDGYNDIPLSLPRGEAQVWTQLLRALEERAVWVLRDVAQVPADRSAYAGARAAA
RLSLTDPLVAERAGTDESALSQGRGQDLLSTVSIRYSGHSLAEVGRVYLFLQPRGPHA
LGGPSGRGQVLVFLGQSEGLRSRPSQVDDAVVRFQYYEDSSGKSILYVVDVSFNRLT
SLPLGALRGLGELQELYLKGNELKTLPPGLLTPTPKLEKLSLWLEDRTPEALLQVRAAS
PSLWVDARGHGLTALPALPARTRHLLLESTKKTIPELDQPPKLRGVLQGHLESSRNDP
FLHRNHIRSIAPGAFDRLPNLSSLTLSRNHLAFLPEQPSRLQDPVLVVSRLSGLWPVSE
ARVLEDRPLSDKGSGDSSQVTQVSPQRIALRLRLVPLRAWLAGRPERAPYRDLERAR
AKWDTANNPLYKEATSTFPALRGRLLPYLAEDELRAAAAPGRLSRNKITHLPGALLDKI
VLLEQERARAKWDTHYAQSLRKWNQPVNLSRLRYLGVTLSPRLSALPQKLAAALEHH
HHHH

Peptides (marked red) with 6 amino acid residues flanking sequences on both sides (marked black) from 6 proteins listed below were randomly concatenated in GP1 sequence. 7 Cys and 2 Met were replaced with Ala and Ile, respectively (marked blue)

in the flanking sequences. 6 x His tag and linker sequence (marked orange) was from expression vector.

**Integrin alpha-IIb (500 000 to 800 000 copies/platelet) (single-pass)**
1. LAEVGRVYLFLQPRGPHALG
2. TQLYGRFGSAIAPLGDLDRDGYNDI
3. GGPSGRGQVLVFLGQSEGLRSRPSQV
4. PLSLPRGEAQVWTQLLRALEERA
5. PEQPSRLQDPVLVVSRLSGLWP

**Integrin beta-3 (500 000 to 800 000 copies/platelet) (single-pass)**
1/2. PVSEARVLEDRPLSDKGSGDSSQVTQVSPQRIALRLR
3/4. SCMGLKIGDTVSFSIEAKVRGCPQEKEKSFTIKPVGFKDSLIVQ
5. DDCVVRFQYYEDSSGKSILYVV
6. ERARAKWDTANNPLYKEATSTF
7. ERARAKWDTHYAQSLRKWNQPV

**platelet glycoprotein Ib alpha chain (60 000 copies/platelet) (single-pass)**
1/2/3. DVSFNRLTSLPLGALRGLGELQELYLKGNELKTLPPGLLTPTPKLEKLSL
4/5. LESTKKTIPELDQPPKLRGVLQGHLESSRNDPFLH
6. ALSQGRGQDLLSTVSIRYSGHSL

**platelet glycoprotein Ib beta chain (60 000 copies/platelet) (single-pass)**
1. RLVPLRAWLAGRPERAPYRDL
2. PALRGRLLPYLAEDELRAACAPG
3. ARAAARLSLTDPLVAERAGTDES
4. AVWVLRDVAQVPADRSAYCGA

**platelet glycoprotein IX (60 000 copies/platelet) (single-pass)**
1. LWVDCRGHGLTALPALPARTRHLLL
2. LWLEDRTPEALLQVRCASPSL

**platelet glycoprotein V (60 000 copies/platelet) (single-pass)**
1. RLSRNKITHLPGALLDKMVLLEQ
2/3. HRNHIRSIAPGAFDRLPNLSSLTLSRNHLAFL
4. NLSRLRYLGVTLSPRLSALPQ

**GP2.** Number of amino acids: 566; Molecular weight: 64 983.79; Theoretical pI: 9.66; Grand average of hydropathicity (GRAVY): -0.579.

MSATYKGKRSIYAVFESDVNLKGIPVYRRPESKATNATLDPRSFLLRNPNDKYEPFWE
DEEKNESGLTEYRLVSINKNFLEKFVQGLDIGPTKTQVGLIQYANNPRVVFNLNTYKTK
EEIITSDFRIGFGSFVEKTVIPYILENGKLSEGVTISYKSYAKNGQKTIKKQVVLEEGTIAF
KNWVKTGLQFAKRLQVNLLVKPSEKIQVLKNLVNLKIPLLYDAEIHLTRSTNINFLVLRLR
SGEPQTFTLKFKRAEDINPLKIGQTSSSVSFKSENFRHDNILRFGIAVLGYLNRNALDTK
LSLTNKGEVFNELVGKQRISGNYHYSTKAYWNISRKYAQNRYTDLVAIQNKNEIDYLNK
VLPYYSSYYWIGIRKNNKTWTWVGTKKALTNEKINAKWDTGENPIYKSAVTTVLQAQT
RYGFDQFALYKEGDPAPERWYRASPIITVTAAHSGTYRAYSFSSLPLTRKSNGGQDG
GRQDVHSREQVGTKVNVTVEDERTLVRRNNTFLSLRDVFGKDLIYTLYYWKSSSSGK
KTAKTNTNEFLIDVDKGENYAFKLAAALEHHHHHH

Peptides (marked red) with 6 amino acid residues flanking sequences on both sides (marked black) from 7 proteins listed below were randomly concatenated in GP1 sequence. 5 Cys and 3 Met were replaced with Ala and Ile, respectively (marked blue) in the flanking sequences. 6 x His tag and linker sequence (marked orange) was from expression vector.

**platelet glycoprotein 4 (20 000 copies/platelet) (multi-pass)**
1. QKTIKKQVVLEEGTIAFKNWVKTG
2. LQFAKRLQVNLLVKPSEKIQVLKN
3. TYKGKRSIYAVFESDVNLKGIPVYR

**P-selectin (10 000 copies/platelet) (single-pass)**
1/2/3/4/5.
YHYSTKAYWNISRKYCQNRYTDLVAIQNKNEIDYLNKVLPYYSSYYWIGIRKNNKTWTWVGTKKALTNE

**Integrin beta-1 (2 000 to 4 000 copies/platelet) (single-pass)**
1. LVLRLRSGEPQTFTLKFKRAED
2. ITSDFRIGFGSFVEKTVMPYI
3. LSLTNKGEVFNELVGKQRISGN
4. ILENGKLSEGVTISYKSYCKNG
5. EKMNAKWDTGENPIYKSAVTTV

**Integrin alpha-2 (2 000 to 4 000 copies/platelet) (single-pass)**
1/2/3. KNFLEKFVQGLDIGPTKTQVGLIQYANNPRVVFNLNTYKTKEEMI
4. HDNILRFGIAVLGYLNRNALDTK
5. NLVNLKIPLLYDAEIHLTRSTNINF
6. DINPLKIGQTSSSVSFKSENFRH

**Proteinase-activated receptor 1 (PAR-1) (2 500 copies/platelet) (multi-pass)**
1/2/3. RRPESKATNATLDPRSFLLRNPNDKYEPFWEDEEKNESGLTEYRLVSINK

**Platelet glycoprotein VI (1 000 copies/platelet) (single-pass)**
1. LQCQTRYGFDQFALYKEGDPAP
2. PERWYRASPIITVTAAHSGTYRCYSFSS
3. LPLTRKSNGGQDGGRQDVHSR

**Tissue factor (single-pass and secreted)**
1/2/3/4.
EQVGTKVNVTVEDERTLVRRNNTFLSLRDVFGKDLIYTLYYWKSSSSGKKTAKTNTNEFLIDVDKGENYCF

**PLin.** Number of amino acids: 585; Molecular weight: 67 004.32; Theoretical pI: 9.53; Grand average of hydropathicity (GRAVY): -0.216.

MSLRELKTILPLINFKVSSAKFAPTQEKVTFEIYDPNLAFLRFVVYEEPDIIKQVLVENFSNFTNRIASGLEKLLGPKNSEGLHSARDVPVVAILGSGGGRAIVGFSILFNRVLGVSGSQSRGSTIEELLALLKWYSTSAFSRLEKLGLFQTSRLILIGETIKIVIEDYHFKLKFDPELLFNKQFQYQNPNKNRDELNGFFNKLIRNVIGTKGKKQLPDAQLLARRFLLRRLFQTTRLILIGETIKIVIEEYFLQLKFDPELLFGVQFQYRNRIAIEGLLEVKLTLLHVLHKFRFQAADYGYKS

WEAFSNLSYYTRALPPVPPAVQKVSQASIDQSRQIKYQSPDEFERIYEPLDVKSKKIHV
LGSLARGILDLNTYNVVKAPQGKNLLTHGRWFWEFVNATFIREILIRIKENVGKAWAED
VLALVKHPLTANKLPFPKIDPYVFDREGLKEAYPKGQRVDSSNYDPFRLWLAGSYIQPT
KFVSFEFSAQKNRSYVISSFTELKAYDLLSKASVQFVDYNKRQISRIDPELIKTVTINASS
SRSGLDDIQGLIDKYEPSGINAQRGQLSPEKLAAALEHHHHHH

Peptides (marked red) with 6 amino acid residues flanking sequences on both sides (marked black) from 6 proteins listed below were randomly concatenated in PLin sequence. 4 Cys and 9 Met were replaced with Ala and Ile, respectively (marked blue) in the flanking sequences. 6 x His tag and linker sequence (marked orange) was from expression vector.

**Thromboxane-A synthase (CYP5A1) (multi-pass)**
1. LLALLKWYSTSAFSRLEKLGL
2. EPDMIKQVLVENFSNFTNRMASGLE
3. LPNKNRDELNGFFNKLIRNVI
4. GLLEVKLTLLHVLHKFRFQAC

**Prostaglandin G/H synthase 1 (COX-1) (peripheral)**
1. LLTHGRWFWEFVNATFIREMLMRI
2. GTKGKKQLPDAQLLARRFLLRR
3. LFQTTRLILIGETIKIVIEEY (common with COX-2)
4. YFLQLKFDPELLFGVQFQYRNRIAME

**Cytosolic phospholipase A2 (cytoplasmic vesicles)**
1/2. KLLGPKNSEGLHSARDVPVVAILGSGGGRAMVGFS
3. SILFNRVLGVSGSQSRGSTMEE
4. PDEFERIYEPLDVKSKKIHV
5. KLPFPKIDPYVFDREGLKEC

**1-phosphatidylinositol 4,5-bisphosphate phosphodiesterase gamma-2 (exosomes)**
1. SLRELKTILPLINFKVSSAKF
2. LGSLCRGILDLNTYNVVKAPQGKN
3. YPKGQRVDSSNYDPFRLWLCGS
4. APTQEKVTFEIYDPNLAFLRFVVYEE

**1-phosphatidylinositol 4,5-bisphosphate phosphodiesterase beta-2 (exosomes)**
1. KENVGKAWAEDVLALVKHPLTAN
2. QGLIDKYEPSGINAQRGQLSPE
3/4/5. YIQPTKFVSFEFSAQKNRSYVISSFTELKAYDLLSKASVQFVDYNKRQMSRI

**Prostaglandin G/H synthase 2 (Cox-2) (peripheral)**
1. ADYGYKSWEAFSNLSYYTRALPPVP
2. LFQTSRLILIGETIKIVIEDY (common with COX-1)
3. YHFKLKFDPELLFNKQFQYQN
4. PPAVQKVSQASIDQSRQMKYQS
5. DPELIKTVTINASSSRSGLDDI

**PLout.** Number of amino acids: 637; Molecular weight: 71 339.29; Theoretical pI: 9.69; Grand average of hydropathicity (GRAVY): -0.307.

MAARSNRVTVFPIGIGDRYDAAQLRILAGPAGDSNVVKLQRIEDFYASRLLDLVFLLDG
SSRLSEAEFEVLKAFVVDIPYASKGLYLETEAGYYKLSGEAYGFVARIDGSGNFQVLLS
DRYFNKTAAFNDRVYATYQVTRGLASLNAEEIKSQFEGFVKDIILNKAISAHRYSGVVYP
LKSLGRLKSTDPVKAAQFEPPGRQIIAIRSVTVRLPGLHNSLVKLKHGAGSFEIQKGDQ
NPQIAAHVISEASSKTTSVLQWAEKGYYTISTPSYRITSASVNDSGEYRAQRGLSGRSD
PIQLEIHRGWLLLQVSSRVFTEGEPLALRAHAWKDKLVYNVLYYRNGKAFKFQIRSKSN
FIIFLKNTVISDKPLVKVTFFQNGKSQKFSHIKNSLKVAAISESPVAEKSASTTYNKKYQY
YSNKHARGSTPFHFARIPYTLSQTRDVFDATFEAAHRAVSPLPYLRNARYDVRAIAIKT
TSGIHPKNIQSLEVIGKGTHANQALAVKTTSQVRPRHITSLEVIKAGPHAPDPQHERIITV
STNGSIHSPRFPHTYPFGRKSRVVDLNLLTEEVRLYSATPIKITFKSDDYFVAKPGFKIY
YSLLDALDKKIAEFDTVEDLLKYFNPESKLAAALEHHHHHH

Peptides (marked red) with 6 a.a. residues flanking sequences on both sides (marked black) from 12 proteins listed below were randomly concatenated in PLout sequence. 16 Cys and 6 Met were replaced with Ala and Ile, respectively (marked blue) in the flanking sequences. 6 x His tag and linker sequence (marked orange) was from expression vector.

**Low affinity immunoglobulin gamma Fc region receptor II-a (single-pass)**
1. DKPLVKVTFFQNGKSQKFSH
2. STDPVKAAQFEPPGRQMIAIR

**P2Y purinoceptor 12 (multi-pass)**
1. FQIRSKSNFIIFLKNTVISD
2. PFHFARIPYTLSQTRDVFDCT

**P2Y purinoceptor 1 (multi-pass)**
1. CISAHRYSGVVYPLKSLGRLK
2. CAFNDRVYATYQVTRGLASLN

**Prostaglandin F2-alpha receptor (multi-pass)**
1. IKNSLKVAAISESPVAEKSAST

**CD40 ligand (single-pass)**
1. NCEEIKSQFEGFVKDIMLNK
2/3. SFEMQKGDQNPQIAAHVISEASSKTTSVLQWAEKGYYTMS

**Phospholipase A2, membrane associated (peripheral, cell membrane)**
1. TTYNKKYQYYSNKHCRGST

**Von Willebrand factor (secreted)**
1/2/3. MPYASKGLYLETEAGYYKLSGEAYGFVARIDGSGNFQVLLSDRYFNKTC
4. RSVTVRLPGLHNSLVKLKHGAG
5. FEACHRAVSPLPYLRNCRYDV
6/7. DFYCSRLLDLVFLLDGSSRLSEAEFEVLKAFVVDM
8/9. AARSNRVTVFPIGIGDRYDAAQLRILAGPAGDSNVVKLQRIED

**Platelet basic protein (secreted)**
1/2. RCMCIKTTSGIHPKNIQSLEVIGKGTHCNQ

**Platelet factor 4 (secreted)**

1.QCLCVKTTSQVRPRHITSLEVIKAGPHCP

**Platelet-derived growth factor C (secreted)**

1. DPQHERIITVSTNGSIHSPRFPHTYP
2. FGRKSRVVDLNLLTEEVRLYSCTP

**Platelet-derived growth factor D (secreted)**

1. IKITFKSDDYFVAKPGFKIYYSLL
2. DALDKKIAEFDTVEDLLKYFNPES

**High affinity immunoglobulin gamma Fc receptor I (single-pass)**

1/2/3/4/5.
STPSYRITSASVNDSGEYRCQRGLSGRSDPIQLEIHRGWLLLQVSSRVFTEGEPLALR
CHAWKDKLVYNVLYYRNGKAFK
